# Fermented soshiho-tang with *Lactobacillus plantarum* enhances the antiproliferative activity in vascular smooth muscle cell

**DOI:** 10.1186/1472-6882-14-78

**Published:** 2014-02-28

**Authors:** Jung-Jin Lee, Hyeeun Kwon, Ji-Hye Lee, Dong-Gun Kim, Sang-Hyuk Jung, Jin Yeul Ma

**Affiliations:** 1 Korean Medicine (KM)-Based Herbal Drug Development Group, Korea Institute of Oriental Medicine, 1672 Yuseong-daero, Yuseong-gu, Daejeon 305-811, Republic of Korea; 2 Department of Pharmacology, Chungnam National University, College of Pharmacy, Daejeon 305-764, Republic of Korea

**Keywords:** Soshiho-tang, Fermentation, Vascular smooth muscle cells, Antiproliferative effect

## Abstract

**Background:**

Soshiho-tang (SST) is a traditional medicine widely used for the treatment of chronic hepatitis. SST has been shown to confer a variety of pharmacological activities, including prevention of hepatotoxicity, promotion of liver regeneration, and modulation of liver fibrosis. In this study, we investigated the antiproliferative activity of native and fermented (FSST) formulations of SST in vascular smooth muscle cells (VSMCs) and examined the potential underlying mechanisms driving these effects.

**Methods:**

SST, along with preparations fermented with *Lactobacillus plantarum* KFRI-144 (S-A144), *L. amylophilus* KFRI-161 (S-A161) and *L. bulgaricus* KFRI-344 (S-A344), were investigated to determine their effects on the proliferation and viability of VSMCs, along with the signalling pathways underlying these effects.

**Results:**

S-A144 exhibited a strong, dose-dependent inhibition of VSMC proliferation relative to untreated controls, but the others did not affect. In addition, S-A144 significantly decreased the phosphorylation of Akt and PLCγ1 in a dose-dependent manner and induced cell cycle arrest at the G_0_/G_1_ phase characterised by decreased expression of CDKs, cyclins and PCNA.

**Conclusions:**

The findings suggest that S-A144 exhibit enhanced inhibition of PDGF-BB-induced VSMC proliferation comparison to S-AOR through the suppression of cell cycle progression and expression of cell cycle-related proteins, along with the downregulation of Akt phosphorylation.

## Background

Abnormal proliferation and migration of vascular smooth muscle cells (VSMCs) are key events in the pathogenesis of vascular proliferative diseases, such as atherosclerosis and restenosis [1-3]. Following vascular injury, abnormal VSMCs transition to a proliferative phenotype characterised by increased expression of cell cycle and proliferation genes [4]. The cell cycle is a common point of convergence for the mitogenic signalling cascades, which consists of four distinct sequential phases (G_0_/G_1_, S, G_2_ and M). Major check points are controlled by multiple protein kinases, such as cyclin component and a catalytic cyclin-dependent kinase (CDK) [4,5].

One of the principal regulators of migration and subsequent proliferation in VSMCs is platelet-derived growth factor (PDGF)-BB, which is secreted by VSMCs and endothelial cells following injury [6-8]. Signalling through the PDGF receptor is mediated through interactions with multiple SH2 domains including phospholipase-C (PLC) γ1, phosphatidylinositol 3-kinase (PI3K) and Ras/Raf-1 [9]. PDGF-BB activates the extracellular-regulated kinases (ERK) via Ras/Raf-1, and Akt via PI3K [10,11], triggering downstream signal transduction and cell cycle progression [12,13]. As ERK1/2 and Akt are the major signal transduction proteins involved in the regulation of proliferation and differentiation [14-16], these pathways are important for the development of vascular disease by VSMCs.

Soshiho-tang (SST) is a traditional medicine widely used for the treatment of chronic hepatitis [17,18]. SST has been shown to confer a variety of pharmacological activities, including prevention of hepatotoxicity, promotion of liver regeneration and modulation of liver fibrosis [19-21]. However, the antiproliferative activity of SST in VSMCs thus far has not been explored.

Fermentation with pharmaceutical fungal species has been shown to improve the therapeutic effect of some herbal medicines [22-24]. Fermentation has been suggested to increase the concentration of bioactive components, such as antioxidants, reduce the risk of ethanol-induced liver toxicity and improve the anti-inflammatory activity of many compounds [2,23,25]. In this study, we investigated the antiproliferative activity of SST in VSMCs in both fermented (FSST) and unfermented forms.

## Methods

### Materials

Ginseng Radix, Pinellia Tuber, Bupleurum Root, Glycyrrhizae Radix et Rhizoma, Scutellaria Root, Zingiberis Rhizoma Crudus and Zizyphi Fructus were purchased from Yeongcheon herbal market (Yeongcheon, Korea). Identification of all plant material was confirmed by Prof. Ki Hwan Bae of the College of Pharmacy, Chungnam National University (Daejeon, Korea), and all voucher specimens were deposited in the herbal bank in Korea Institute of Oriental Medicine (KIOM, Daejeon, Korea). Dulbecco’s Modified Eagle Medium (DMEM) was purchased from Lonza (Wakersville, MD, USA). Fetal bovine serum (FBS) and phosphate-buffered saline (PBS) were purchased from Hyclone (Longan, UT, USA). Penicillin/streptomycin and trypsin/EDTA were purchased from Gibco (Grand Island, NY, USA). Anti-phospho-ERK1/2, anti-phospho-Akt, anti-phospho-PLCγ1, anti-ERK1/2, anti-Akt, anti-PLCγ1, anti-CDK2, anti-CDK4, anti-cyclin D_1_, anti-cyclin E_1_ and anti-β-actin antibodies were from Cell Signaling Technology Inc. (Beverly, MA). Anti- phospho-proliferating cell nuclear antigen (PCNA) was purchased from Abfrontier (Seoul, Korea). PDGF-BB was obtained from Upstate Biotechnology (Lake Placid, NY, USA). Cell Counting Kit-8 (CCK-8) was purchased from Dojindo Molecular Technologies (Rockville, MD, USA). Other chemicals were of analytical grade.

### Preparation of SST extract

SST was prepared according to previously reported method [19]. Briefly, 1674.5 g medicinal herbal drug, including Bupleurum Root 600 g, Glycyrrhizae Radix et Rhizoma 100 g, Ginseng Radix 200 g, Pinellia Tuber 200 g, Scutellaria Root 400 g, Zingiberis Rhizoma Crudus 74.5 g and Zizyphi Fructus 100 g, was decocted with 16.745 L of boiling water in stainless oven for 3 h at 115°C using a Gyeongseo Extractor Cosmos-600 (Incheon, Korea), and then the decoction was filtered using standard testing sieves (150 μm; Retsch, Haan, Germany). Then, the filtrate was lyophilized and stored in desiccators at 4°C. For the fermentation of SST extract, the freeze-dried extract powder was then dissolved in distilled water, and kept at 4°C. In addition, for the experiment of this study, the freeze-dried extract powder was then dissolved in 50% dimethyl sulfoxide (DMSO, v/v with phosphate buffered saline) and filtered (pore size, 0.2 μm), and kept at 4°C (S-OR).

### Fermentation of SST extract

In this study, *Lactobacillus plantarum* KFRI-144 (S-A144), *Lactobacillus amylophilus* KFRI-161 (S-A161) *and Lactobacillus bulgaricus* KFRI-344 (S-A344) used with the fermentation of SST was derived from Korea Food Research Institute (KFRI, Seongnam-si, Korea). Two successive transfers of the test organisms in MRS broth (10 g/L peptone, 10 g/L beef extract, 5 g/L yeast extract, 20 g/L glucose, 1 mL/L Tween 80, 2 g/L K_2_HPO_4_, 5 g/L sodium acetate, 2 g/L triammonium citrate, 0.2 g/L MgSO_4_∙7H_2_O, 0.2 g/L MnSO4∙4H_2_O, pH 6.2-6.6) for lactobacilli culture at 37°C for 24 h, and then the activated cultures were again inoculated into broth. It was properly diluted to obtain an initial population of 1–5 × 10^6^ CFU/mL and served as the inoculum. The viable cell count of strain was determined in duplicate by using the pour-plate method on MRS agar. In fermentation process, 5 mL of SST was inoculated with 0.05 mL of the inocula as above, and then this was incubated at 37°C for 48 h. At an interval of 24 h, fermented SSTs were collected and were analyzed pH. *Lactobacillus plantarum* KFRI-144 (S-A144), *Lactobacillus amylophilus* KFRI-161 (S-A161) *and Lactobacillus bulgaricus* KFRI-344 (S-A344) were selected as the high acid-production using pH analysis and 1st screening test of antiproliferative activity.

### Cell culture

Rat aortic VSMC were purchased from BioBud (Seongnam, Korea), which was isolated by enzymatic dispersion as previously described [26,27]. VSMC was cultured in DMEM, supplemented with 10% FBS, 100 IU/mL penicillin, 100 μg/mL streptomycin, 8 mM HEPES and 2 mM L-glutamine at 37°C in a humidified atmosphere of 95% air and 5% CO_2_ incubator. The purity of VSMC culture was confirmed by immunocytochemical localization of α-smooth-muscle actin. The passage number of VSMC used in this experiment was with 5–7*.*

### Cell proliferation assay

VSMC was measured by both direct counting and nonradioactive colorimetric WST-1 assay (CCK-8, Takara, Japan). For direct cell counting, rat aortic smooth muscle cells were seeded into 12-well culture plates at 4×10^4^ cells/mL, and then cultured in DMEM containing 10% FBS at 37°C for 24 h. After reaching at ~70% of confluence, the cells were incubated with serum-free medium for 24 h, treated with SST (S-OR) and FSST formulas (S-AOR, S-A144, S-A161, and S-A344) for another 24 h in newly fresh serum-free medium and stimulated by PDGF-BB (25 ng/mL). After 24 h the cells were trypsinized by trypsin-EDTA and counted by using hemocytometer under microscopy. For nonradioactive colorimetric WST-1 assay, all experimental procedures were performed as recommended by manufacturer’s instructions, and the results were expressed as percentage of PDGF-BB-stimulated control.

### Cell viability assay

VSMC was seeded into 96 well culture plates at 3×10^4^ cells/mL, and then cultured in DMEM containing 10% FBS at 37°C for 24 h. After reaching at 70% of confluence, the cells were incubated with serum-free medium for 24 h. The cells were exposed to 500 μg/mL S-A144 or 50 μM digitonin as a cytotoxic control at various times. WST-1 reagent was added to the medium, and the cells were incubated for an additional 2 h. The absorbance was measured at 450 nm using a spectrophotometer.

### Cell cycle progression analysis

The measurement of cell cycle progression was performed as previously described [26,27]. The assay condition was the same as described in the section of cell proliferation assay. After being stimulated by PDGF-BB (25 ng/mL) for 24 h, cells were trypsinized and centrifuged at 1,500 × *g* for 7 min. The centrifuged pellets were suspended in 1 mL of 1 × PBS, washed twice, and fixed with 70% ethanol for 48 h. The fixed cells were briefly vortexed and centrifuged at 15,000 × *g* for 5 min. The ethanol was discarded and the pellets were stained with 500 μL propidium iodide (PI) solution (50 μg/mL PI in sample buffer containing 100 μg/mL of RNase A). Before flow cytometry analysis, each sample was incubated at room temperature for 1 h. The PI-DNA complex in each cell nucleus was measured with FACScalibur (Becton & Dickinson Co.). The individual nuclear DNA content was reflected by fluorescence intensity of incorporated PI. The rate of the cell cycle within G_0_/G_1_, S and G_2_/M phase was determined by analysis with Modfit LT software.

### Immunoblotting assay

Immunoblotting assay was performed as previously described [26,27]. Rat aortic smooth muscle cells were stimulated with PDGF-BB (25 ng/mL) for 5 min for ERK 1/2 and PLCγ1, 15 min for Akt phosphorylation assays. For the assay of CDK2, CDK4, cyclin D_1_, cyclin E_1_ and PCNA expressions, VSMC were stimulated by PDGF-BB (25 ng/mL) for 24 h. The detected proteins were normalized by β-actin or respective total proteins, respectively. The intensities of bands were quantified using a Scion-Image for Window Program (Scion Corporation, MA).

### Statistical analysis

Data were expressed as means ± S.E.M. Statistical comparisons were conducted via one-way analysis of variance (ANOVA) followed by Dunnett’s test to determine which groups differed significantly from the control group. Comparison of the two groups was conducted via an unpaired Student’s *t* test (GraphPad, San Diego, USA). A p value of < 0.05 was considered significant.

## Results

### Effects of SST and FSST on VSMC proliferation

**Figure 1:**
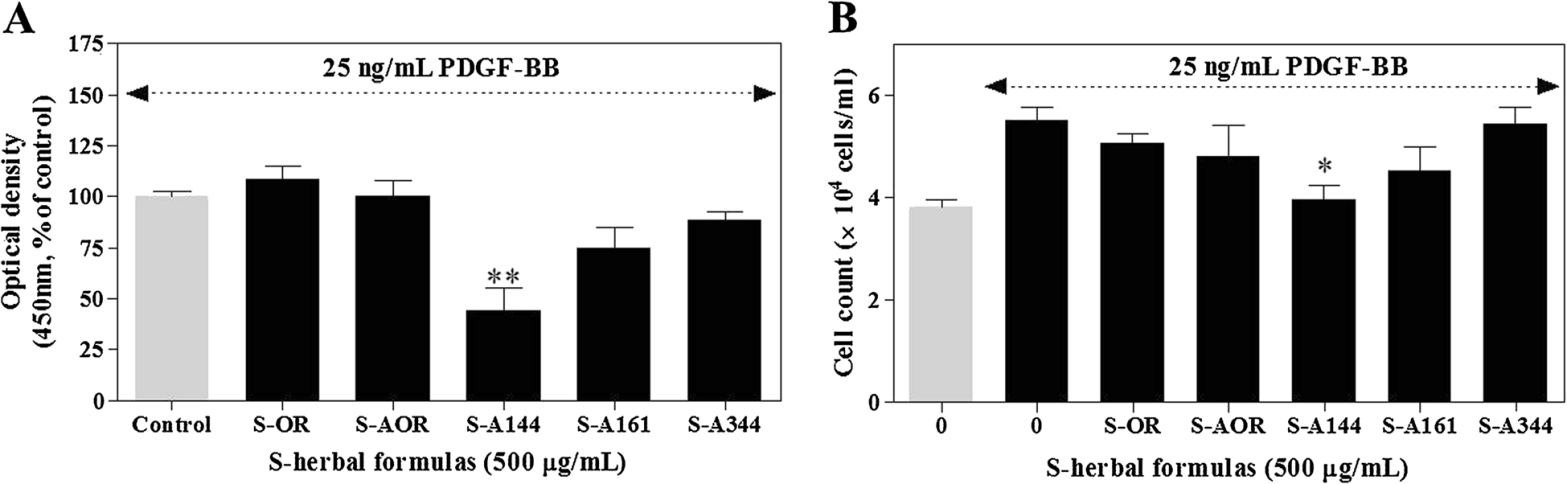
**Effects of SST and FSST formulas on VSMC proliferation and viability.** VSMCs were seeded into 12-well plates at 4 × 10^4^ cells/mL and cultured in DMEM containing 10% FBS at 37°C for 24 h. The cells were then incubated in serum-free medium for 24 h, treated with SST (S-OR) or FSST formulas (S-AOR, S-A144, S-A161 and S-A344) for another 24 h in fresh serum-free medium and stimulated by PDGF-BB (25 ng/mL). **(A)** Cell proliferation was determined using a colourimetric WST-1 assay read at 450 nm (*n* = 4); **(B)** cells were counted using a haemocytometer (*n* = 3). Values are expressed as the mean ± S.E.M. Statistically significant differences relative to PDGF controls (PDGF-stimulated) are indicated by *P < 0.05 and **P < 0.01.

To compare the antiproliferative effects of SST formulas on VSMCs, we performed colourimetric WST-1 and cell counting assays. Among the FSST formulas, SST fermented with *Lactobacillus plantarum* KFRI-144 (S-A144) exhibited the strongest inhibition of PDGF-BB-induced proliferation in VSMCs (Figure 1A). This effect was stronger than that of S-AOR, a sterilised formulation of SST.

**Figure 2:**
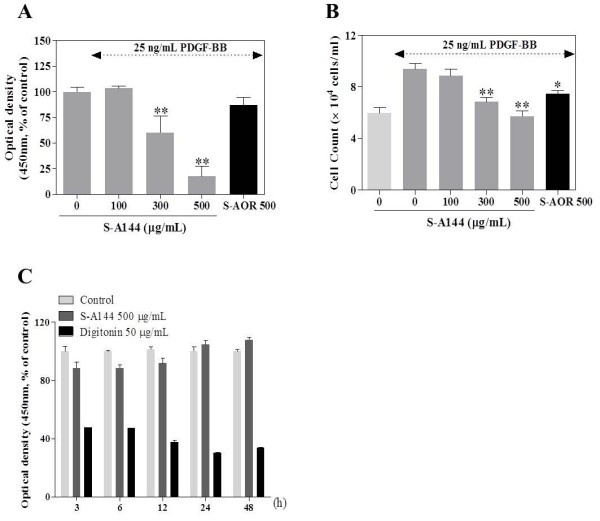
**Effect of S-A144 on VSMC proliferation and viability.** VSMCs cultured in serum-free medium were stimulated with 25 ng/mL PDGF-BB for 24 h, and the effect of various concentrations of S-A144 (100–500 μg/mL) or S-AOR (500 μg/mL) on cell proliferation and viability was measured. **(A)** Cell proliferation was determined using a colourimetric WST-1 assay read at 450 nm (*n* = 4). **(B)** Cells were counted using a haemocytometer (*n* = 3). **(C)** VSMCs were treated with 500 ng/mL S-A144 for 24 h; cell viability was determined using a WST-1 assay read at 450 nm (*n* = 4). Digitonin was used as a positive cytotoxic control. Values are expressed as mean ± S.E.M. Statistically significant differences relative to PDGF controls (PDGF-stimulated) are indicated by *P < 0.05 and **P < 0.01.

In cell counting assays, treatment of VSMCs with 25 ng/mL PDGF-BB significantly increased cell proliferation after 24 h (Figure 1A). Pretreatment of cells with 500 μg/mL S-A144 significantly reduced VSMC proliferation to 4.0 ± 0.3 × 10^4^ cells/well (Figure 1B). Further analysis of compound S-A144 alone showed a concentration-dependent inhibition of VSMC proliferation (Figure 2A), with cell numbers decreased significantly to 8.9 ± 0.5 (100 μg/mL), 6.8 ± 0.4 (300 μg/mL) and 5.7 ± 0.4 × 10^4^ cells/well (500 μg/mL) compared with 9.4 ± 0.4 × 10^4^ cells/well for PDGF-BB treatment controls (Figure 2B). These antiproliferative effects were not due to enhanced cytotoxicity, as even the highest concentration of S-A144 (500 μg/mL) exhibited no cytotoxicity in serum-free medium (Figure 2C). In contrast, 50 μg/mL digitonin as a positive cytotoxic control was cytotoxic [26,28].

### Effects of S-A144 on ERK1/2, Akt and PLCγ1 activation

**Figure 3:**
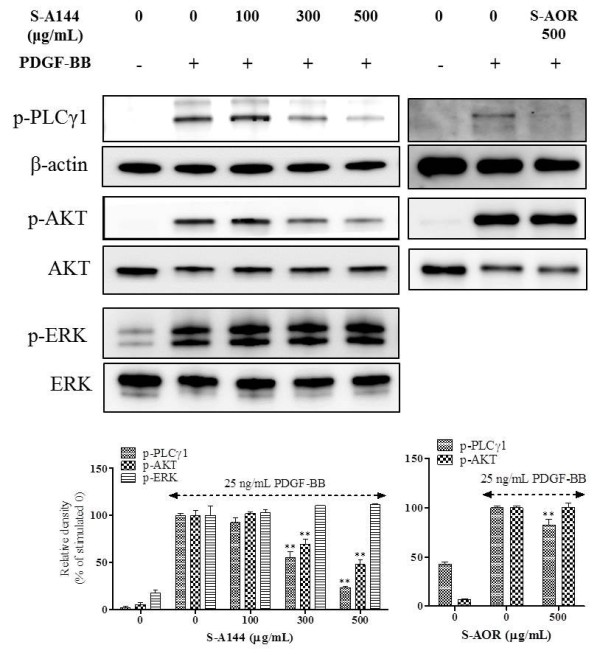
**Effects of S-A144 on the PDGF-induced activation of ERK1/2, Akt and PLCγ1.** Quiescent VSMCs cultured in serum-free medium were stimulated with 25 ng/mL PDGF-BB, followed by the addition of various concentrations of S-A144 (100–500 μg/mL) or S-AOR (500 μg/mL) to determine the effect on phosphorylation of ERK1/2, Akt and PLCγ1, as measured by SDS-PAGE and immunoblotting. Total ERK1/2, Akt or β-actin were used for normalisation. Data are representative of three independent experiments. Values are expressed as the mean ± S.E.M. Statistically significant differences relative to PDGF controls (PDGF-stimulated) are indicated by **P < 0.01.

Our previous study demonstrated that early signal, such as Akt, ERK1/2 and PLCγ1 phosphorylation, is important signal transduction in hyper-proliferation of VSMCs [26,29]. Hence, to investigate the role of early signalling events in the antiproliferative activity of S-A144, phosphorylation of Akt, ERK1/2 and PLCγ1 was measured in VSMCs following stimulation with PDGF-BB. As shown Figure 3, S-A144 significantly decreased the phosphorylation of Akt and PLCγ1 in a concentration-dependent manner, but ERK1/2 phosphorylation was unaffected. The inhibitory effect of S-A144 on Akt phosphorylation was significantly greater than that seen with S-AOR (48.0 ± 4.7% and 100.2 ± 4.2%, respectively). These results indicate that the antiproliferative action of S-A144 derived by inhibition of Akt and PLCγ1 phosphorylation, the activity enhancement of S-A144 comparison with S-AOR was due to the suppression of PI3K-mediated signalling pathway.

### Effect of S-A144 on cell cycle progression

**Figure 4:**
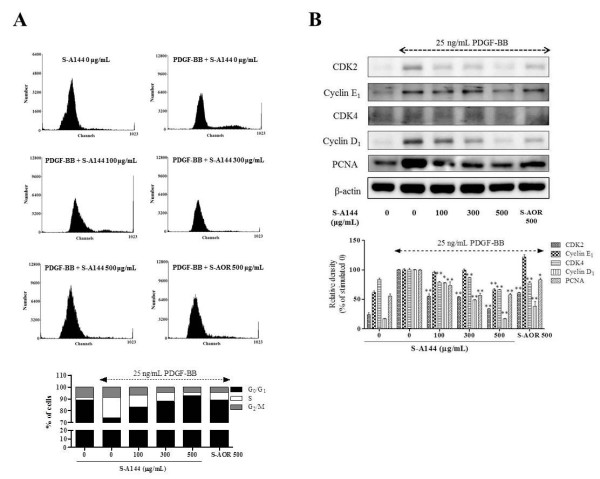
**Effects of S-A144 on cell cycle progression and cell cycle-related protein expression in PDGF-BB-stimulated VSMCs.** VSMCs cultured in serum-starved medium were stimulated with 25 ng/mL PDGF-BB, followed by the addition of various concentrations of S-A144 (100–500 μg/mL) or S-AOR (500 μg/mL) to determine the effects on DNA synthesis. **(A)** Cell cycle progression data are representative of three independent experiments. **(B)** CDKs, cyclins and PCNA expression were measured by SDS-PAGE and immunoblotting; total β-actin was used for normalisation. Immunoblots were analysed by densitometry; values indicate the level of expression relative to controls. Data are representative of three independent experiments. All values are expressed as the mean ± S.E.M.

We next examined the effects of PDGF-BB and S-A144 on cell cycle progression. The addition of PDGF-BB to VSMCs cultured in serum-free media resulted in considerable synchronisation in the G_0_/G_1_ phase (74.2 ± 1.8%); another 17.0 ± 2.0% of the cells were in S phase (Figure 4A). Following treatment with S-A144, the percentage of cells in G_0_/G_1_ phase increased in a dose dependent manner, ranging from 83.3 ± 1.9 (100 μg/mL) to 92.9 ± 0.8% (500 μg/mL), respectively. Taken together, these results show that the antiproliferative effects of S-A144 result in the arrest of cells in G_0_/G_1_ phase through the inhibition of specific signalling pathways, including Akt and PLCγ1.

### Effect of S-A144 on cell cycle-related protein expression

Cell cycle progression is strictly regulated through the expression of cell cycle-related proteins, such as CDK2, CDK4, cyclin D1, cyclin E1 and PCNA [5]. To demonstrate the mechanism of S-A144-induced the arrest of cell cycle, we investigated the effect of S-A144 on CDK2, CDK4, cyclin D1 and cyclin E1 expression. The result shown in Figure 4B represented that S-A144 inhibited the expression of CDK 2, CDK4 and cyclin D_1_ in a concentration-dependent manner. In the effect of S-A144 on cyclin E_1_ expression, S-A144 only inhibited at a concentration of 500 μg/mL, however, S-AOR at the same concentration did not affect. Moreover, in other cell cycle-related protein expression, S-A144 was greater than S-AOR. In addition, expression of PCNA, synthesised as a phosphorylated retinoblastoma (Rb) protein-mediated gene product in early G_0_/G_1_ and S phase, was also inhibited by S-A144 (Figure 4B). This effect was significantly greater for S-A144 than S-AOR, suggesting that the enhanced antiproliferative effects of S-A144 compared to S-AOR occur via arrest in G_0_/G_1_ phase through inhibition of cell cycle-related protein expression.

## Discussion

This study demonstrated that fermentation of SST enhanced the antiproliferative effects of this compound on VSMCs. This enhanced effect occurred via arrest in the G_0_/G_1_ phase through inhibition of Akt phosphorylation and cell cycle-related protein expression.

Cardiovascular disease is a complex condition stemming from a variety of physiological processes, including VSMC proliferation, hypertension and inflammation [30,31]. Among these causes, VSMC proliferation plays a central role in the pathogenesis of atherosclerosis and restenosis after vascular injury, and possibly in the development of hypertension [4,32]. For the clinical application, research on the VSMC proliferation has been report in connection with restenosis, such as the development of bio-stent, the suppression of neointima formation after balloon injury, and the importance of VSMC proliferation after vascular injury and stenting [33-35]. Controlling VSMC proliferation may therefore be important for the treatment of cardiovascular disorder and atherosclerosis [26,36].

Fermentation has recently been shown to confer beneficial effects on VSMC proliferation, including inhibition of proliferation and migration of SMCs by Chungtaejeon, a Korean fermented tea, and the vasoprotective effects mediated by the nonalcoholic constituents of red wine [37,38]. To identify the mechanism by which fermentation enhanced the antiproliferative activity of SST, we investigated a variety of SST fermentation formulas including eight strains of *Lactobacillus* and two strains of *Bifidobacterium* (data not shown) compared with S-AOR, a sterilised formulation of SST. From these preliminary studies, we selected three strains of *Lactobacillus* that exhibited the strongest effect on SST antiproliferative activity. In Figure 1, we describe several SST fermentation formulas, with S-A144 exhibiting the strongest antiproliferative effect on VSMCs.

S-A144 significantly inhibited PDGF-BB-induced VSMC proliferation in a dose-dependent manner (Figure 2). Moreover, Akt and PLCγ1 phosphorylation were identified as possible molecular mechanisms by which S-A144 inhibited cell proliferation.

PDGF-mediated cellular proliferation is a highly regulated process involving PLCγ1, PI3K and mitogen activated protein (MAP) kinase activation [39,40]. PLCγ1 phosphorylation modulates the downstream signal transduction of a variety of growth factors, including PDGF [9]. S-AOR significantly inhibited PDGF-BB-induced PLCγ1 phosphorylation, but did not inhibit AKT phosphorylation. These data therefore indicate that PLCγ1 may be a target of S-AOR in VSMCs. In contrast, S-A144 showed a greater inhibitory effect on Akt phosphorylation than S-AOR (Figure 3), indicating that fermentation-related products were modulating Akt activity.

Akt, a serine/threonine protein kinase, is phosphorylated through the PI3K pathway and is important in regulating cell cycle progression [11], which is modulated by regulatory factors, including cyclin and CDKs, with pRb considered an important inhibitor of proliferation [5,10].

VSMC proliferation is modulated primarily by regulation of the cell cycle, S-A144 inhibited cell cycle progression by arresting cells in G_0_/G_1_ phase (Figure 4A). This tightly regulated temporal progression is controlled by the sequential activation of CDKs and their subunits, cyclins that phosphorylate the Rb protein. S-A144 also inhibited the cell cycle-related protein involving CDKs, cyclins, and PCNA expression (Figure 4B), which is synthesised as a pRb phosphorylation-mediated gene product required for the G_0_/G_1_ to S phase transition [41], consistent with the effects seen on cell cycle progression. These effects were greater for S-A144 than S-AOR, suggesting that S-A144 may exhibit enhanced inhibition of cell cycle progression and expression of cell cycle-related proteins via the inhibition of Akt phosphorylation.

## Conclusions

This study demonstrates that S-A144, an SST formulation fermented with *L. plantarum*, exhibit enhanced inhibition of PDGF-BB-induced VSMC proliferation comparison to S-AOR through the induction of cell cycle arrest at the G_0_/G_1_ phase and inhibition of CDKs, cyclins and PCNA expression. This inhibition may be mediated through a downregulation of Akt phosphorylation. Together, these data suggest that S-A144 may be useful in the prevention of atherosclerosis and restenosis.

## Abbreviations

PI3K: Phosphatidylinositol 3′-kinase; CCK-8: Cell counting kit-8; CDK: Cyclin-dependent kinase; DMEM: Dulbecco’s modified Eagle’s medium; DMSO: Dimethyl sulphoxide; ERK: Extracellular signal-regulated kinase; FBS: Foetal bovine serum; PBS: Phosphate-buffered saline; PCNA: Phospho-proliferating cell nuclear antigen; PDGF: Platelet-derived growth factor; PLCγ1: Phospholipase C-γ1; SDS-PAGE: Sodium dodecyl sulphate-polyacrylamide gel electrophoresis; SH: Soshiho-tang; VSMC: Vascular smooth muscle cells.

## Competing interests

The authors declare that they have no competing interests.

## Authors’ contributions

JJL and JYM participated in the design of the study; JJL, HK, JHL, DGK and SHJ performed all experiments; JJL and JYM analysed the data and wrote the paper. All authors read and approved the final manuscript prior to submission.

## Pre-publication history

The pre-publication history for this paper can be accessed here:

http://www.biomedcentral.com/1472-6882/14/78/prepub
